# Reconstructed colorectal cancer model to dissect the anti-tumor effect of mesenchymal stromal cells derived extracellular vesicles

**DOI:** 10.1186/s40164-024-00526-2

**Published:** 2024-06-18

**Authors:** Edoardo D’Angelo, Sarah Tassinari, Andrea Biccari, Sara Crotti, Francesca Sensi, Asia Marangio, Ombretta Repetto, Giuseppe Corona, Linda Bellucci, Federica Antico, Federico Caicci, Gaya Spolverato, Giovanni Montini, Benedetta Bussolati, Marco Agostini, Federica Collino

**Affiliations:** 1 General Surgery 3, Department of Surgery, Oncology and Gastroenterology, University of Padova, via Giustiniani 2, Padua, 35128 Italy; 2 NanoInspired biomedicine lab, Fondazione Istituto di Ricerca Pediatrica Città della Speranza, Padua, Italy; 3 Department of Medical Sciences, University of Turin, Turin, 10126 Italy; 4 Department of Women and Children’s Health, University of Padova, via Giustiniani 2, Padua, 35128 Italy; 5 Immunopathology and Cancer Biomarkers, CRO Aviano, National Cancer Institute, IRCCS, Aviano, 33081 Italy; 6 Laboratory of Translational Research in Paediatric Nephro-urology, Fondazione IRCCS Ca’ Granda-Ospedale Maggiore Policlinico, Milano, Italy; 7 Pediatric Nephrology, Dialysis and Transplant Unit, Fondazione IRCCS Ca’ Granda-Ospedale Maggiore Policlinico, Milano, Italy; 8 Department of Biology, University of Padova, Padua, 35131 Italy; 9 Department of Clinical Sciences and Community Health, University of Milano, Milan, Italy

**Keywords:** Mesenchymal stromal cells, Extracellular vesicles, 3D model, Colorectal cancer

To the editor,

Colorectal cancer (CRC) represents one of the leading causes of oncological-related death in both sexes worldwide [1]. A better understanding of CRC biology is urgently needed to reduce its progression. Recently the crucial role of the extracellular matrix (ECM) and extracellular vesicles (EVs) in maintaining the tumor pathophysiology has been recognized [2, 3]. Dysregulation of ECM remodeling has been shown to contribute significantly to tumor fate, for example by inducing hypoxia followed by metabolic changes and drug resistance [4]. In parallel, mesenchymal stromal cell-derived EVs (MSC-EVs) can play dual roles in tumor growth and progression [5, 6], and their effects on CRC are still debated. Here, the capability of MSC-EVs to diffuse into tumor ECM and be uptaken by engrafted tumor cells was evaluated using a three-dimensional (3D) CRC model. Moreover, the role of MSC-EVs in influencing tumor growth as well as their effects on the ECM was analyzed by proteomic analysis.

We used the ECM from CRC patients obtained by decellularization (Supplementary material 1) [7]. The ECM structure was characterized before (DM) and after repopulation (RM) with CRC cells, HT29 (Supplementary material 2A-B). ECM components were uniformly maintained in RM, as observed by PAS and Ki67-positive staining (Supplementary material 2C-D). We proposed using this model to evaluate the MSC-EV role as therapeutics for CRC treatment.

The potential use of MSC-EVs as a bioactive compound with per se anti-tumor activity is dependent on their incorporation capability that we established in normal culture conditions (Supplementary material 2E). Then, we explored the MSC-EV diffusion into the ECM using DiI-labeled EVs. DiI-labelled-EVs were captured within the RM and accumulated in the cytoplasm of the HT-29 (Fig. 1A). EVs presented a diameter of 50–150 nm, consistent with a small EV population (Supplementary material 3A-B). Together with the classical exosomal and mesenchymal surface markers (Supplementary material 3C), we identified 379 proteins within the EVs by proteomic analysis (Supplementary material 4). The gene-ontology enrichment analysis highlighted cargo proteins related to DNA regulation and ECM organization (Supplementary material 3D-F). Interestingly, MSC-EVs were also identified in DM-EV in the absence of cancer cells (not shown), supporting the EV capability to migrate inside the ECM as described by [8].

**Fig. 1:**
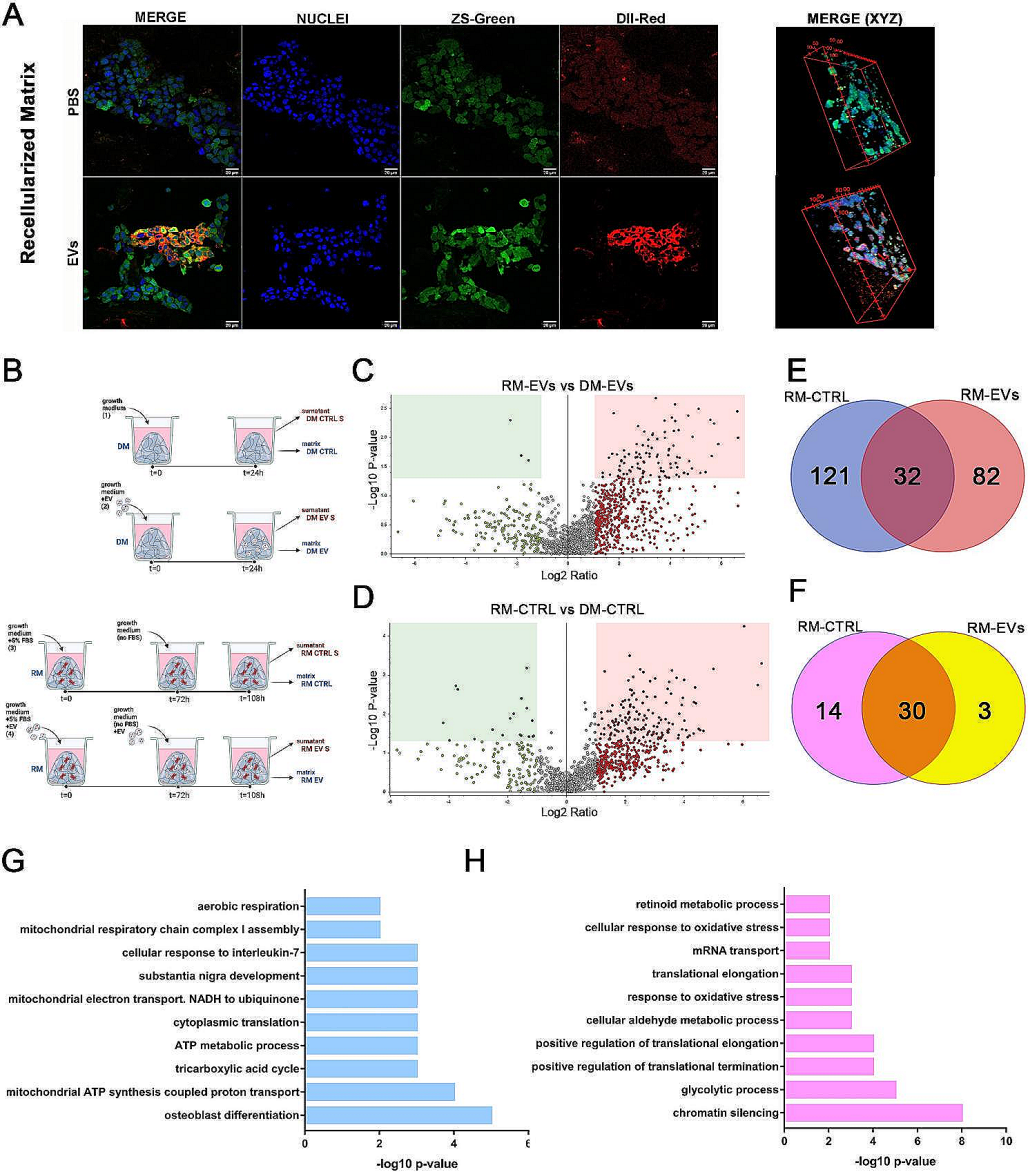
MSC-EVs active uptake in the 3D-CRC model and proteome analysis of DM and RM samples. **(A)** Representative immunofluorescence images of CRC biopsies repopulated with ZSGreen-HT29 CRC cell line incubated with EVs-DiL or PBS-DiL. Cell nuclei were counterstained using DAPI. Scale bar=20 μm (left panel). 3D reconstruction of EVs-DiL or PBS-DiL (red) diffusion in decellularized CRC biopsies repopulated with ZSGreen-HT29 CRC cell line (green, right panel; scale bar = 20 μm). **(B)** Schematic representation of proteome and secretome analysis in DM and RM samples in which differentially abundant proteins were investigated in DM (upper panel) or RM (lower panel) after EV-treatment or not. Matrix samples were divided into four groups: *(i)* decellularized CRC biopsies, EV-untreated (DM-CTRL) or *(ii)* EV-treated (DM-EV); *(iii)* decellularized CRC biopsies repopulated with HT29 cancer cell line, EV-untreated (RM-CTRL) or *(iv)* decellularized CRC biopsies repopulated with HT29 cancer cell line EV-treated (RM-EV). Supernatants *(S)* of each condition (labelled in red) were also collected. Volcano plots of the comparison between the proteomic profile of RM and DM after incubation with MSC-EVs **(C)** or control medium **(D)**; up-regulated proteins (red squares) and down-regulated proteins (green squares) between RM-EV vs. DM-EV and RM-CTRL vs. DM-CTRL. Venn diagrams of exclusively up-regulated **(E)** or down-regulated **(F)** proteins in RM-CTRL and RM-EVs groups. Up-regulated proteins specific of CTRL group are labelled in blue, those specific of EVs treated-group are in red and the up-regulated proteins shared by the two groups are in dark red. Down-regulated proteins specific of CTRL group are labelled in pink, those specific of EVs treated-group are in yellow and the down-regulated proteins shared by the two groups are in orange. **(G-H)** Functional annotation of the respectively up-regulated proteins in RM-CTRL and RM-EV using DAVID Bioinformatics Resources for Biological processes

In this light, we hypothesized that MSC-EVs can affect cancer cell behavior directly, by their cellular uptake, or indirectly, by modifying the ECM biological properties. Therefore, we compared firstly the proteomic profile of DM and RM after incubation with MSC-EVs or in their absence. (Fig. 1B). We identified a total of 117 differently expressed proteins between RM-EV and DM-EV, while 165 were differently expressed between RM-CTRL and DM-CTRL (Fig. 1C, D). Next, we compared the two lists to discriminate the direct effects of MSC-EVs on HT-29. Considering the up-regulated proteins, 82 and 121 proteins resulted exclusively modulated in EV- or CTRL-treated groups, respectively (Fig. 1E and Supplementary material 5). In the case of down-regulated proteins, 14 were specific for CTRL- and 3 for the EV-treated group (Fig. 1F and Supplementary material 5). The RM-CTRL showed enrichment for proteins involved in the cell cycle and proliferation (Fig. 1G). Conversely, the enriched proteins in RM-EV were involved in gene silencing, translational processes negative regulation, and oxidative stress, suggesting a cell population exposed to stress conditions (Fig. 1H). This led us to speculate that MSC-EVs could prevent CRC cells from homing in the tumor-ECM.

This hypothesis was confirmed by a reduction of the viability of EV-treated CRC cells engrafting the ECM after MSC-EVs administration, mainly due to enhanced apoptosis measured by Tunel assay (Fig. 2A, B) as well as by the downregulation of the anti-apoptotic gene BCL-2 and the upregulation of the pro-apoptotic gene BAK-1 (Fig. 2C). Interestingly, a significant but slighter cytotoxic effect was observed culturing CRC cells in the 2D setting (Supplementary material 2F), highlighting the importance of the direct activity of the EVs towards the ECM. The transcriptional down-regulation of Ki-67, c-Myc, CCND2, and CCNE1, in concomitant with the over-expression of CDKN1A (Fig. 2D), confirmed an antitumor effect of the MSC-EV in the 3D-CRC [9, 10]. Interestingly, the MSC-EV cargo is enriched with molecules involved in DNA synthesis and transcription, and epigenetic gene silencing (Supplementary material 4). A strong overrepresentation of molecules belonging to the complement system was also identified (Supplementary material 3G, H), which can mediate immunosurveillance mechanisms against cancer [11]. The secretome profile of RM-EV was consistent with the proteomic data obtained in the 3D model, showing the enrichment of proteins related to ECM organization compared to RM-CTRL (Fig. 2E-H and Supplementary material 6).

**Fig. 2:**
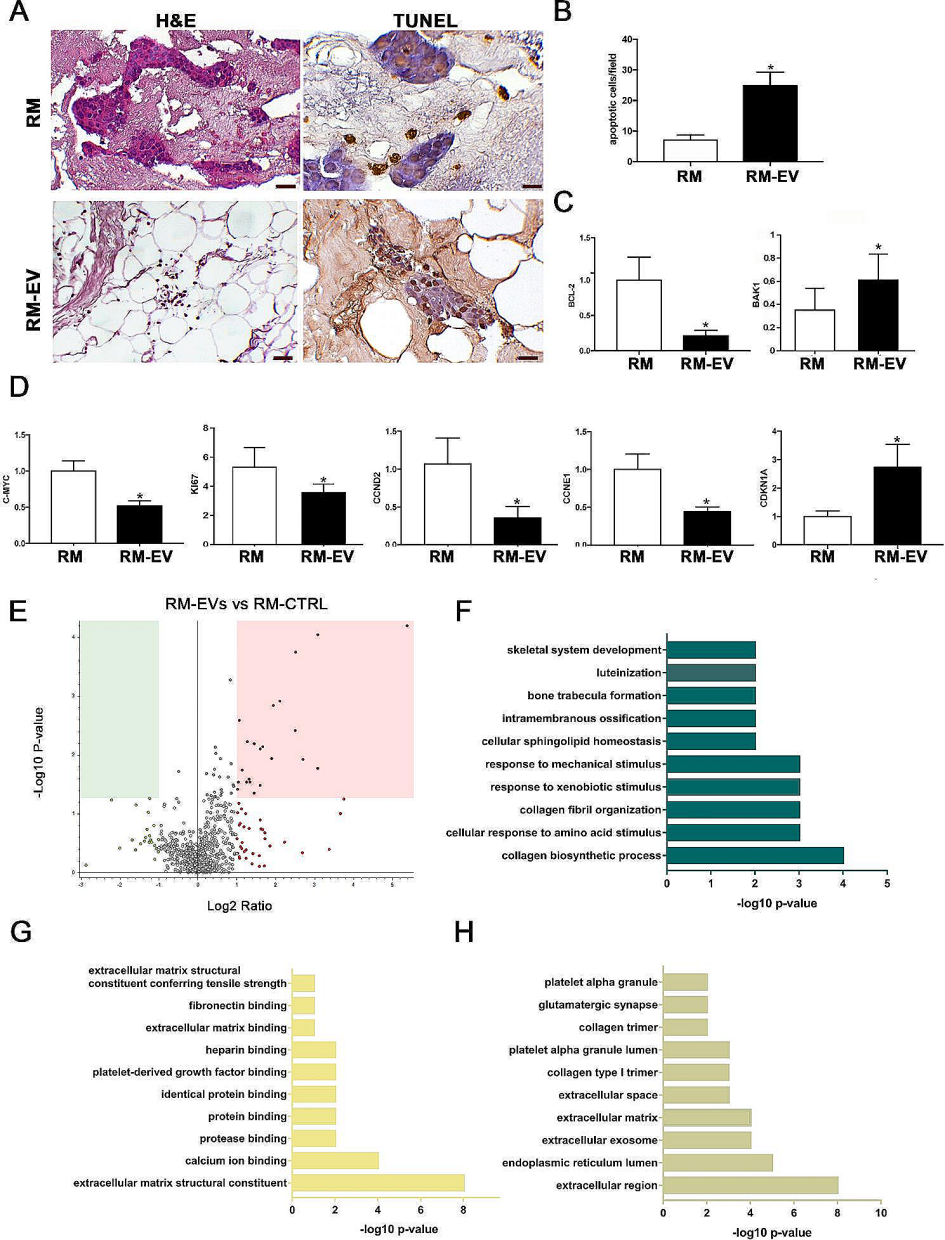
Biological effect on cell cycle and apoptosis of the MSC-EVs treatment in the 3D-CRC model and secretome analysis of RM samples. **(A)** Representative H&E images of 3D-CRC untreated (RM) or treated (RM-EV) with MSC-EVs (left panel). The apoptotic cells were detected using TUNEL assay, the DNA fragmentation is indicated by ApopTag Plus Peroxidase positive staining (brown) (right panel). Scale bar = 20 μm. **(B)** Quantification of apoptotic cells in the 3D-CRC model treated or untreated with MSC-EVs and expressed as apoptotic cells/field. Gene expression level of apoptosis and cell cycle-related genes in the 3D-CRC model treated or untreated with MSC-EVs: **(C)** BCL-2 and BAK1. **(D)** C-MYC, KI-67, CCND2, CCNE1 and CDKN1A. *P*< 0,05 vs. RM, unpaired two-sided Student’s t-test. **(E)** Volcano plots of the comparison between the secretome profile of RM-EV vs. RM-CTRL, The up-regulated proteins (red squares) and down-regulated proteins (green squares) in the RM-EV secretome in respect to the RM-CTRL were defined. The 23 secreted proteins up-regulated in RM-EV were functionally annotated using DAVID Bioinformatics Resources in **(F)** Biological processes, **(G)** Molecular functions and **(H)** Cellular components

In conclusion, we establish a promising assay to investigate the biological activity of MSC-EVs in a 3D environment. In the 3D-CRC model, the direct influence of each biological component studied was discerned, allowing a better definition of the tumor-stroma response to EV treatment. In the future, this model can be translated to other tumors and EV sources to evaluate different anti-cancer strategies in a 3D biomimicking environment.

### Electronic supplementary material

Below is the link to the electronic supplementary material.

Supplementary Material 1

Supplementary Material 2

Supplementary Material 3

Supplementary Material 4

Supplementary Material 5

Supplementary Material 6

## Data Availability

The proteomic data generated in this study are available from the corresponding authors on reasonable request. All other data generated or analyzed during this study are included in this published article (and its supplementary information files).

## Abbreviations

CRC: Colorectal cancer
TME: Tumor microenvironment
ECM: Extracellular matrix
EVs: Extracellular vesicles
MSC: Mesenchymal stem cell
MSC-EVS: Mesenchymal stem cell-derived extracellular vesicles
DM: Decellularized matrix
RM: Recellularized matrix
PAS: Periodic Acid-Schiff
3D-CRC: Three-dimensional patient derived-CRC model
FDR: False discovery rate
GO: Gene Onthology
CTRL: Control
CCND2: Cyclin-D2
CCNE1: Cyclin-E1
CDK: Cyclin Dependent Kinase
