# Supplementary material for: Reconstructed colorectal cancer model to dissect the anti-tumor effect of mesenchymal stromal cells derived extracellular vesicles
Source: Exp Hematol Oncol. 2024 Jun 18;13:61. doi: 10.1186/s40164-024-00526-2 (PMC11184788; doi:10.1186/s40164-024-00526-2)
Supplement: Supplementary file 1 — Supplementary Material 1 [file 40164_2024_526_MOESM1_ESM.docx]

**Reconstructed colorectal cancer model to dissect the anti-tumor effect of mesenchymal stromal cells derived extracellular vesicles.**

Edoardo D'Angelo^1,2^, Sarah Tassinari^3^, Andrea Biccari^1^, Sara Crotti^2^, Francesca Sensi^2,4^, Asia Marangio^1^, Ombretta Repetto^5^, Giuseppe Corona^5^, Linda Bellucci^6,7^, Federica Antico^3^, Federico Caicci^8^, Gaya Spolverato^1^, Giovanni Montini^6,7,9^, Benedetta Bussolati^3^, Marco Agostini^1,2 #^, Federica Collino ^6,7,9 #^

1 General Surgery 3, Department of Surgery, Oncology and Gastroenterology, University of Padova, via Giustiniani 2, 35128 Padua, Italy.

2 NanoInspired biomedicine lab, Fondazione Istituto di Ricerca Pediatrica Città della Speranza, Padua, Italy.

3 Department of Medical Sciences, University of Turin, 10126 Turin, Italy

4 Department of Women and Children’s Health, University of Padova, via Giustiniani 2, 35128 Padua, Italy.

5 Immunopathology and Cancer Biomarkers, CRO Aviano, National Cancer Institute, IRCCS, 33081 Aviano, Italy

6 Laboratory of Translational Research in Paediatric Nephro-urology, Fondazione IRCCS Ca' Granda-Ospedale Maggiore Policlinico, Milano, Italy.

7 Pediatric Nephrology, Dialysis and Transplant Unit, Fondazione IRCCS Ca' Granda-Ospedale Maggiore Policlinico, Milano, Italy.

8 Department of Biology, University of Padova, 35131 Padua, Italy.

9 Department of Clinical Sciences and Community Health, University of Milano, Milan, Italy.

**# Last authors:** Marco Agostini and Federica Collino.

*** Correspondence:** Correspondence should be addressed to Edoardo D’Angelo, Ph.D, email: edoardo.dangelo@unipd.it; and Federica Collino, PhD, [federica.collino@unimi.it](mailto:federica.collino@unimi.it).

**Content:**

Supplementary methods

Figure legends

Supplementary figure legends

Supplementary table legends

**Patient and Methods**

*Patients*

A series of 9 pathologic tissue samples from CRC patients who underwent curative surgery between February 2015 and December 2016 were collected from General surgery 3 at the University Hospital of Padua (Department of Surgery, Oncology and Gastroenterology). This study was conducted according to the principles expressed in the Declaration of Helsinki. Written informed consent was obtained from every enrolled individual and protocol was approved by ethics committee of institution (Ethical Committee Approved Protocol Number: 448/2002). All the patients enrolled fulfilled the following inclusion criteria: histologically confirmed primary adenocarcinoma of the colon, age > 18 years, and written informed consent. Patients with a known history of a hereditary CRC syndrome or undergoing neoadjuvant treatments were excluded.

|  | | **Patients**  **(n=9)** |
| --- | --- | --- |
| **Age** | Median (range), yrs | 65 (39-91) |
| **Sex** | Male  Female | 4 (44)  5 (56) |
| **TNM** | 2  3 | 5 (56)  4 (44) |
| **Grade** | 1  2  3 | 1 (11)  7 (78)  1 (11) |

Clinical-pathological characteristics of CRC patients enrolled in the study. Data are expressed as numbers. TNM: Tumor Node Metastasis stage; (%) unless stated otherwise.

*Cell Cultures*

A human colon adenocarcinoma cell line (HT29) was used in all the experiments. HT29 were grown in Roswell Park Memorial Institute (RPMI) 1640 Medium (EuroClone, Milan, Italy) supplemented with 10% fetal bovine serum (FBS) (HyClone, Pittsburgh, USA), 1 mM glutamine, 10 mg/mL penicillin, and 10 mg/mL streptomycin at 37◦C in a 5% CO2 humidified atmosphere. A lentiviral produced HT29 cell line carried both the ZsGreen fluorescent protein and the firefly luciferase under the EF1-alpha promoter was used for tracking analysis and was a gift from Dr. Luca Urbani (Institute of Hepatology, Foundation for Liver Research, London, UK). MSCs were obtained from Lonza (Lonza, Basel, Switzerland) and cultured in MSC basal medium (MSCBM, Lonza) (1). MSCs were used up to the sixth passage of culture.

*Isolation and characterization of EVs*

EVs were collected from MSCs after overnight starvation with serum-free RPMI-1640 (Lonza). The cell supernatant was collected and centrifuged for two times at 3,000 x g for 20 minutes to remove possible cell debris or apoptotic bodies. Then the supernatant was ultracentrifuged at 100,000 x g for 2 hours (h) at 4°C using a 70ti rotor (Beckman Coulter Optima L-90K ultracentrifuge; Beckman Coulter, Brea, CA, USA). EVs were then resuspended in serum-free RPMI-1640 supplemented with 1% DMSO and stored at -80°C. For EV distribution experiments, labelling was conducted using the Vybrant™ DiI Cell-Labeling Solution (Thermo Fisher) at the final concentration of 1µM. After 1h incubation, 9 mL of PBS were added to the labelled EVs (EVs-DiI) and PBS (PBS-DiI) solution and the resulting final volume of 10 mL was centrifuged at 4000 rpm for 15 minutes with the 100 KDa Amicon filters (Amicon-Merck Millipore, Burlington, MA, USA). Filters were washed twice to eliminate unbound Dye. The eluate was then discarded, and the retained EVs-DiI and PBS-DiI were freshly used. Concentration and size distribution of EVs (mean diameter of 97.76 nm) were determined by Nanosight LS300 system (Malvern Panalytical, Malvern, UK) equipped with a 488 nm laser module (sample dilution 1:100). Briefly, EV preparations were diluted in sterile saline solution and analyzed by the Nanoparticle Analysis System using the NTA 3.2 Analytical Software. 3 videos of 60 seconds at camera level 14 and threshold 5 were captured using a syringe pump 30. The NTA settings were kept constant between samples.

*Bead-based cytofluorimetric analyses*

Bead-based cytofluorimetric analyses was conducted on MSC-EVs with the MACsPlex exosome kit (Miltenyi Biotec, Bergisch Gladbach, Germany). Shortly, 1x10^9^ MSC-EVs were diluted with MACsPlex Buffer to a final volume of 120 µL. A total of 15 µL of MACsPlex exosome capture bead solution, containing beads coupled to 37 exosomal surface epitopes and 2 control isotypes, was added and incubated overnight at 4°C on an orbital shaker (450rpm) protected from light. The day after 15 µL of MACSPlex Exosome Detection Reagent cocktail containing antibodies against tetraspanins (CD9, CD63, CD81) were added and incubated for 1h at room temperature and samples were maintained in rotation. Beads and detection antibodies were incubated in EV resuspension medium without particles and used as a negative control. After washing steps, samples were analyzed by flow cytometry using the BD FACSCelesta flow cytometer (Becton Dickinson, San Diego, Ca, USA), capable of detecting the necessary fluorescence signals.

*A three-dimensional patient derived-CRC model (3D-CRC)*

CRC tissue was obtained at the core of infiltrating neoplasia. The decellularization process was obtained by detergent-enzymatic treatment as previously described (2). Tumor decellularized matrices (DM) were incubated overnight with a growth medium containing Primocin antibiotic (InvivoGen, Kampenhout, Belgium) at 4^◦^C. To normalize the intra-sample variability, scaffolds were cut into comparable dimensions before seeding. All matrices were then injected with 1x10^6^ HT29, resuspended in 10 μL of collagen I (diluted 2:3 with RPMI-1640), using a 30G syringe needle (3,4). Tumor recellularized matrices (RM) were initially incubated for 4h in the incubator at 37°C with 5% CO_2_. The complete medium was then carefully added and changed every two days.

*Colorectal cancer matrix treatment with EVs*

DM were incubated overnight with 1x10^9^ MSC-EVs before the recellularization step. The day after, following the HT29 injection, 3x10^9^ MSC-EV were administered at 24h and then at 72h pos-injection. Five days post-seeding EV-treated or untreated matrices were either formalin-fixed and paraffin-embedded for the IHC staining or fixed in 4% paraformaldehyde (PFA) and then included in 20% glycerol for the immunofluorescence analysis. Matrices were also snap-frozen in liquid nitrogen for molecular analysis.

*Scanning and transmission electron microscopy analyses*

Matrices were fixed with 2% glutaraldehyde in 0.1 M phosphate; following washing they were cut into segments of approximately 1 cm length and cryoprotected in 25 % sucrose, 10 % glycerol in 0.05 MPBS (pH 7.4) for 2h, then fast frozen. At the time of analysis, samples were processed as previously described (2). Images were recorded with a Jeol 7401 FEG scanning electron microscope. Transmission electron microscopy (TEM) was performed also to characterize EVs. One drop of EV solution (about 25 µl) was placed on a 400-mesh holey film grid. After staining with 2% uranyl acetate (for 2 minutes), the sample was observed with a Tecnai G2 (FEI) transmission electron microscope operating at 100 kV. Images were captured with a Veleta (Olympus Soft Imaging System) digital camera.

*Tracking analysis of EVs in 3D-CRC model*

Fluorometric analysis was conducted on matrices treated or not with labelled EVs. Diffusion and distribution of labelled MSC-EVs in DM and RM was examined. Fixed whole matrices were stained with 100 ng/mL 40,6-diamidino-2-phenylindole (DAPI; Sigma-Aldrich) at 4°C for 3h , washed and then included in 20% glycerol in PBS 1X for confocal analysis. Matrices injected with DiI-labelled PBS (PBS-DiI) were used as control. Imaging was performed using a Leica TCS SP5 confocal system (Leica Microsystems S.r.l., Wetzlar, Germany) with a HC PL FLUOTAR 10X (NA 0.3) Dry objective. Series of x-y-z images were collected along the z-axis at 5 µm intervals through-out the sample depth. Three-dimensional reconstruction was performed using “3D Viewer” plugin of Fiji ImageJ software. Moreover, EV presence in RM was also analyzed by global fluorescence intensity (Em. 569 nm) using a microplate reader BioTek Sinergy h1 (AHSI SpA, Bernareggio, MB, Italy).

*Proteome and Secretome analysis*

*Study design*

Proteomics analysis was organized in three parts. In part I of the study, protein cargo of MSC-EV was characterized. In part II of the study, differentially abundant proteins were investigated in DM or RM after EV-treatment or not. Matrix samples were divided into four groups: (i) tumor decellularized matrices, EV-untreated (DM CTRL; n=3) or (ii) EV-treated (DM EV; n=3); (iii) tumor recellularized matrices, EV-untreated (RM CTRL; n=3) or (iv) EV-treated (RM EV; n=3). In part III, the supernatants from RM analyzed in part II were examined searching for differentially abundant proteins.

*Mass spectrometry analysis*

Protein cargo of MSC-EV, DM and RM samples (average weight= 2.4±1.3 mg) and respective supernatants were analyzed by mass spectrometry as indicated below.

*Protein purification and mass spectrometry analysis by LC-MS/MS*

Proteins were extracted from (i) MSC-EV-enriched fractions, (ii) tumor matrices and (iii) supernatants. (i) MSC-EV-enriched fraction (1x10^10^ EV) was reduced to around 50 μL by centrifuging (4,000 x g, 4 °C) in 3,000 molecular weight cut-off concentrators (Vivaspin; Sartorius, Göttingen, Germany). The concentrate was moved to a new tube, and lysed with a lysis buffer (A45735, Thermo Fisher Scientific, Massachusetts, USA) containing protease inhibitors (87785, Thermo Fisher Scientific) and in presence of 0.1% (w/v) RapiGest SF (Waters, Massachusetts, USA). The lysate was subjected to two cycles of freeze (-80 °C) and thaw (4 °C) and sonication (2 x 60 s). The tube was then centrifuged for 20 min at 8,000 x g to remove membrane debris. (ii) Protein extraction from tumor matrices, decellularized or not, was performed according to (Naba A, J Vis Exp. 2015 Jul 23;(101):e53057. doi: 10.3791/53057), starting from 2,4 (± 1,3) mg of sample. (iii) Surnatant proteins were recovered from the serum-free medium, as above described, concentrated to around 50 μL with in 3,000 MW cut-off concentrators (Vivaspin; Sartorius). Protein concentration was measured with the Pierce BCA Protein Assay Kit (Thermo Fisher Scientific). Protein extracts were immediately subjected to further down-stream analyses.

*Isolated* Proteins (100 µg) were reduced, alkylated and digested and peptides cleaned-up with the EasyPep Mini MS Sample Prep Kit (Thermo Fisher Scientific) according to the manufacturer’s protocol. Three biological replicates were analyzed per group. The peptide mixtures were then analyzed with LC-MS/MS, using a Q-Exactive Plus Hybrid Orbitrap mass spectrometer equipped with a UHPLC Vanquish (Thermo Fisher Scientific). Individual samples were analyzed in duplicate. Each tryptic peptide sample was fractionated in a XBridge Peptide BEH C18 column (3.5 µm 2.1 x 150, Waters, Sesto San Giovanni, Milan, Italy) at a flow rate of 200 µL/min using 0.1% formic acid/acetonitrile gradient (eluent A: 0.1% formic acid in ultrapure water; eluent B: 0.1% formic acid in acetonitrile) over a period of 61.5 min, and spray onto the mass spectrometer using an heated electrospray source probe in positive mode (Thermo Fisher Scientific). Acetonitrile, formic acid (FA) and water, all LC-MS grade, were purchased from Sigma Aldrich Srl (Milan, Italy). The mass spectrometer was run in the data-dependent mode with positive polarity at electrospray voltage of 3.52 kV and capillary temperature 325 °C. Full scan MS spectra (m/z 375-1500) were acquired followed by MS/MS scans on the top 10 intense ions, applying a dynamic exclusion window of 30 seconds. Label-free quantification (LFQ) and database search were done with Proteome Discoverer software (version 2.5.0.400) using the Sequest search engine against the human database (UniProt release 2022_02) with the following settings: 1) two max missed cleavage sites allowed, 2) precursor mass tolerance 10 ppm, fragment ion mass tolerance 0.02 Da, 3) cysteine carbamidomethylation as static modification and 4) methionine oxidation as dynamic modification. The false discovery rate (FDR) was set to 0.01. Only proteins identified with a false discovery rate (FDR) medium (5%) or high (1%), a Sequest Score ≥1 and a p-value <0.05 were considered. All potential contaminants coming from culture media were filtered. The abundance ratio (or fold change, FC) of statistically significant proteins was calculated as the ratio of the average LFQ intensities between the two matched groups. Proteins differing in abundance between the two groups were defined as those with a FC≥2 (FC Log2≥1) (proteins increasing in abundance) or a FC≤2 (FC Log2≤-1) (proteins decreasing in abundance).

*Proteins functional annotation*

The functional annotation of differentially abundant proteins (p <0.05) was done with DAVID 6.8 Gene Ontology (GO) biological processes associated with the proteins were evaluated (p <0.01, Fisher’s exact test). Proteins identified in the MSC-EVs were searched for the top 100 proteins released by ExoCarta (<http://exocarta.org>). A qualitative comparison of identified proteins was performed with Venn diagram [http://bioinformatics.psb.ugent.be/webtools/Venn/], to evidence proteins either shared by the interrogated group or unique.

*Immunohistochemistry and immunofluorescence*

Tissue samples were formalin fixed and paraffin embedded. The tissue sections (5 µm thick) were stained with Haematoxylin & Eosin (H&E; Bio Optica), Periodic Acid Schiff (PAS; Bio Optica), anti-KI67 (1:100, Abcam) according to manufacturer's instruction. Immunohistochemical staining was automatically performed using the Bond Polymer Refine Detection kit in the BOND-MAX system (Leica Biosystems). Apoptosis was evaluated using the TUNEL assay (ApopTag Plus Peroxidase in Situ Apoptosis Detection Kit, Millipore) as described by the provider but modifying the digestion time to 5 minutes. For immunofluorescence analysis, cells were incubated with primary antibody against Phalloidin (1:100, Sigma), then slides were washed and incubated with labelled Alexa Fluor secondary antibody (1:200, Thermo Fisher Scientific). Finally, nuclei were counterstained with fluorescent mounting medium plus DAPI. For each specimen, random pictures were collected with a direct microscope.

*Quantitative real time PCR*

RM were weighed, then cut into smaller pieces and digested using Proteinase K (5 mg/ml) for 10 minutes at 37°C. After incubation, Trizol LS (Thermo Fisher Scientific) was added, and the samples were homogenized using 0,15 µl latex beads (Tissue Lyser II, Qiagen). Total RNA was isolated using the miRNeasy MINI kit (Qiagen) according to the manufacturer’s protocol. Total RNA (150 ng) was reverse-transcribed, and levels of specific transcripts were assessed by quantitative real time PCR (qRT PCR). Primers listened below were purchased from MWG-Biotech (Eurofins Scientific, Brussels, Belgium). Five ng of cDNA, 200 nM of specific primers and 1X SYBR Green PCR Master Mix were run on a StepOne Real Time System and data were analyzed by Quant Studio 7 Pro Design and Analysis Software (all from Thermo Fisher Scientific). The amplification reaction was conducted in a final volume of 20 μl using 4 μl of cDNA, TaqMan ® Universal PCR Master Mix 1X (Applied Biosystems) and specific TaqMan ® Gene Expression Assay 1X (Applied Biosystems): Hs04260396_g1 for KI-67 and Hs00832876_g1 for BAK1. Glyceraldehyde-3Phosphate Dehydrogenase (GAPDH) expression was used to normalize

| **Gene Symbol** | **Name** | **FW** | **RW** |
| --- | --- | --- | --- |
| **h-BLC2** | B-Cell Leukemia/Lymphoma 2 | F-CCGGGAGATCGTGATGAAGT | R-ATCCCAGCCTCCGTTATCCT |
| **h-CCND1** | Cyclin D1 | F-TATTGCGCTGCTACCGTTGA | R-CCAATAGCAGCAAACAATGTG  AAA |
| **h-CCND2** | Cyclin D2 | F-GAGCTGCTGGAGTGGGAACT | R-TGCTTGCGGATCAGAGACAG |
| **h-CCNE1** | Cyclin E1 | F-GCCTTGAATTTCCTTATGGTATAC  TTG | R-CTGATACCCTGAAACCTTTTG  CA |
| **h-CDKN1A** | Cyclin-Dependent Kinase inhibitor 1A | F-AGCATGACAGATTTCTACCACTCC  A | R-AGACACACAAACTGAGACTAA  GGCA |
| **h-CDKN2A** | Cyclin-Dependent Kinase inhibitor 2A | F-CCGATCCAGGTCATGATGATG | R-CAGCACCACCAGCGTGTC |
| **h-CDKN2B** | Cyclin-Dependent Kinase inhibitor 2B | F-GCGATCCAGGTCATGATGATG | R-AGCACCACCAGCGTGTCC |
| **h-C-MYC** | Cellular Myelocytomatosis | F-CAGCGACTCTGAGGAGGAACA | R-TGAGGAGGTTTGCTGTGGC |
| **h-GAPDH** | Glyceraldehyde-3-Phosphate Dehydrogenase | F-TGGAAGGACTCATGACCACAGT | R-CATCACGCCACAGTTTCCC |

the cDNA inputs. Below the complete list of primers used in this study is summarized.

*MSC-EV treatment and Cytotoxicity Assay*

CRC cell line were seeded in a 96-well tissue culture plates at 1 × 10^4^ cells/well. After 24 h, cells were incubated with a dose of 2x10^4^ MSC-EVs/cell. After 72h of continuous exposure, 20 µl of resazurin dye (Abcam) were added to the wells and incubated for 2 hours. Fluorescence was read at 530/590 nm using the Tecan Microplate Reader Spark (Tecan LifeScience). Cytotoxicity was determined as the percentage of fluorescence in exposed cells compared to the untreated cells (mean ± SD).

*Statistical analysis*

All graphs and statistical analysis were performed using GraphPad Prism Software v.6. Data are expressed as means ± SD. For comparison between coupled experimental groups two-sided Student’s t-tests (for parametric dataset) and Mann-Whitney test (for non-parametric dataset) were used. One-way ANOVA with Bonferroni's post-test (for parametric dataset) and Kruskal-Wallis’s test with Dunn’s post-test (for non-parametric dataset), was performed for multiple comparisons. A p-value < 0.05 was considered statistically significant.

**Bibliography**

1. Naba A, Clauser KR, Hynes RO. Enrichment of Extracellular Matrix Proteins from Tissues and Digestion into Peptides for Mass Spectrometry Analysis. J Vis Exp. 2015 Jul 23;(101):e53057. doi: 10.3791/53057.

2. D’Angelo E, Natarajan D, Sensi F, Ajayi O, Fassan M, Mammano E, et al. Patient-Derived Scaffolds of Colorectal Cancer Metastases as an Organotypic 3D Model of the Liver Metastatic Microenvironment. Cancers (Basel). 2020 Feb;12(2).

3. Piccoli M, D'Angelo E, Crotti S, Sensi F, Urbani L, Maghin E, Burns A, De Coppi P, Fassan M, Rugge M, Rizzolio F, Giordano A, Pilati P, Mammano E, Pucciarelli S, Agostini M. Decellularized colorectal cancer matrix as bioactive microenvironment for in vitro 3D cancer research. J Cell Physiol. 2018 Aug;233(8):5937-5948. doi: 10.1002/jcp.26403. Epub 2018 Feb 27. PMID: 29244195.

4. Sensi F, D'Angelo E, Piccoli M, Pavan P, Mastrotto F, Caliceti P, Biccari A, Corallo D, Urbani L, Fassan M, Spolverato G, Riello P, Pucciarelli S, Agostini M. Recellularized Colorectal Cancer Patient-derived Scaffolds as in vitro Pre-clinical 3D Model for Drug Screening. Cancers (Basel). 2020 Mar 13;12(3):681. doi: 10.3390/cancers12030681. PMID: 32183226; PMCID: PMC7140024.

**Figure Legends**

**Figure 1:** MSC-EVs active uptake in the 3D-CRC model and proteome analysis of DM and RM samples. **(A)** Representative immunofluorescence images of CRC biopsies repopulated with ZSGreen-HT29 CRC cell line and incubated with EVs-DiL or PBS-DiL. Cell nuclei were counterstained using DAPI. Scale bar=20µm (left panel). 3D reconstruction of EVs-DiL or PBS-DiL (red) diffusion in decellularized CRC biopsies repopulated with ZSGreen-HT29 CRC cell line (green, right panel; scale bar = 20 µm). **(B)** Schematic representation of proteome and secretome analysis in DM and RM samples in which differentially abundant proteins were investigated in DM (upper panel) or RM (lower panel) after EV-treatment or not. Matrix samples were divided into four groups: *(i)* decellularized CRC biopsies, EV-untreated (DM-CTRL) or *(ii)* EV-treated (DM-EV); *(iii)* decellularized CRC biopsies repopulated with HT29 cancer cell line, EV-untreated (RM-CTRL) or *(iv)* decellularized CRC biopsies repopulated with HT29 cancer cell line EV-treated (RM-EV). Supernatants *(S)* of each condition (labelled in red) were also collected. Volcano plots of the comparison between the proteomic profile of RM and DM after incubation with MSC-EVs **(C)** or control medium **(D)**; up-regulated proteins (red squares) and down-regulated proteins (green squares) between RM-EV vs DM-EV and RM-CTRL vs DM-CTRL. Venn diagrams of exclusively up-regulated **(E)** or down-regulated **(F)** proteins in RM-CTRL and RM-EVs groups. Up-regulated proteins specific of CTRL group are labelled in blue, those specific of EVs treated-group are in red and the up-regulated proteins shared by the two groups are in dark red. Down-regulated proteins specific of CTRL group are labelled in pink, those specific of EVs treated-group are in yellow and the down-regulated proteins shared by the two groups are in orange. **(G-H)** Functional annotation of the respectively up-regulated proteins in RM-CTRL and RM-EV using DAVID Bioinformatics Resources for Biological processes.

**Figure 2:** Biological effect on cell cycle and apoptosis of the MSC-EVs treatment in the 3D-CRC model and secretome analysis of RM samples. **(A)** Representative H&E images of 3D-CRC untreated (RM) or treated (RM-EV) with MSC-EVs (left panel). The apoptotic cells were detected using TUNEL assay, the DNA fragmentation is indicated by ApopTag Plus Peroxidase positive staining (brown) (right panel). Scale bar = 20 μm. **(B)** Quantification of apoptotic cells in the 3D-CRC model treated or untreated with MSC-EVs and expressed as apoptotic cells/field. Gene expression level of apoptosis and cell cycle-related genes in the 3D-CRC model treated or untreated with MSC-EVs: **(C)** BCL-2 and BAK1. **(D)** C-MYC, KI-67, CCND2, CCNE1 and CDKN1A. P< 0,05 vs RM, unpaired two-sided Student’s t-test. **(E)** Volcano plots of the comparison between the secretome profile of RM-EV vs RM-CTRL. The up-regulated proteins (red squares) and down-regulated proteins (green squares) in the RM-EV secretome in respect to the RM-CTRL were defined. The 23 secreted proteins up-regulated in RM-EV were functionally annotated using DAVID Bioinformatics Resources in **(F)** Biological processes, **(G)** Molecular functions and **(H)** Cellular components.

**Supplementary material legends**

**Supplementary material 2: (A)** Schematic representation of 3D-CRC model development: 1. Decellularized CRC biopsies were uniformly sized using a punch; 2. pre-rinsed in culture medium for 2h in incubator; 3. cell line was injected using a 30G syringe needle; 4. dry 3D-CRC was cultivated in the incubator for 4-6h and finally 5. Culture medium was added. **(B)** Left panels, immunofluorescence analysis of decellularized CRC biopsy before (DM) and after repopulation with ZSGreen-labelled HT29 CRC cell line (RM) Scale bar=100µm. Right panels, Scanning electron microscopy analysis of decellularized CRC biopsy before (DM) and after repopulation with ZSGreen-labelled HT29 CRC cell line (RM). Scale bar=10µm. **(C)** Patients-derived ECM was analyzed before and after the decellularization process. Hematoxylin and Eosin (H&E) and PAS histology and Collagen IV immunostaining of CRC samples before (Fresh CRC) and after decellularization process (DET CRC) confirmed the successful decellularization and preservation of tissue structures. Scale bar: 100µm. **(D)** Histology and immunohistochemistry staining of 3D-pCRC model: Hematoxylin and Eosin (H&E, upper panel), Periodic acid–Schiff (PAS, middle panel) and KI-67 marker of proliferation, confirmed the successful repopulation of CRC patients-derived ECM, Scale bar=100µm. **(E)** Internalization of MSC-EVs in CRC cells in 2D setting (left panel, Phalloidin in green; middle panel, EVs-DiI in red and right panel, merge). Cell nuclei were counterstained using DAPI. Scale bar=100µm **(F)** Evaluation of the anti-tumor activity of MSC-EVs *in vitro* in a CRC cell line. MSC-EVs were used a dose of 2x10^4^/cell. Data were expressed as mean ± SD).

**Supplementary material 3: (A)** EVs isolated from MSC were characterized for their size and shape. Nanoparticle Tracking assay was used for the analysis of the size distribution of MSC-EVs. **(B)** Representative image of isolated MSC-EVs performed by transmission electron microscopy, scale bar=100 nm. **(C)** Multiplex flow cytometry analysis of EVs markers, such as tetraspanins and mesenchymal markers in three different EV preparation from two MSC lines. The x-axis shows the protein marker profile, whereas the y-axis represents the normalized Median APC fluorescence intensity. Functional annotation using DAVID Bioinformatics Resources of 379 EV proteins identified using mass spectrometry: **(D)** Biological processes; **(E)** Molecular functions; **(F)** Cellular complement. **(G)** Heatmap of Complement and complement-related proteins identified in two different preparations of MSC-EVs. The relative abundance of proteins belonging to the Complement pathway were calculated as the intensity of the precursor ion peak **(H)** Complement and complement-associated protein interaction as predicted from STRING database.

**Supplementary material 4:** List of proteins identified after Label-free untargeted LC-MS/MS-based analysis of MSC EVs and functional annotation in Biological Process (BP), Molecular Function (MF) and Cellular Component (CC) using DAVID Bioinformatics Resources.

**Supplementary material 5:** List of proteins identified after Label-free untargeted LC-MS/MS-based analysis of respectively DM-CTRL, DM-EV, RM-CTRL, and RM-EV and functional annotation in Biological Process (BP), Molecular Function (MF) and Cellular Component (CC) using DAVID Bioinformatics Resources. The modulated proteins between EV-RM and -DM; and CTRL-RM and -DM were underlined. The up-regulated proteins resulted exclusively in CTRL-RM or in EV-RM were highlighted in blue or red, respectively. The down-regulated proteins resulted exclusively modulated in CTRL-RM or EV-RM groups were highlighted in yellow and pink, respectively.

**Supplementary material 6:** List of proteins identified after Label-free untargeted LC-MS/MS-based analysis in the supernatant of respectively DM-CTRL, DM-EV, RM-CTRL, and RM-EV and functional annotation in Biological Process (BP), Molecular Function (MF) and Cellular Component (CC) using DAVID Bioinformatics Resources. The proteins resulted upregulated in EV-RM S in respect to CTRL-RM S were highlighted in red, while the ones downregulated were labeled in green.
