# Supplementary figures and images for: Reconstructed colorectal cancer model to dissect the anti-tumor effect of mesenchymal stromal cells derived extracellular vesicles
Source: Exp Hematol Oncol. 2024 Jun 18;13:61. doi: 10.1186/s40164-024-00526-2 (PMC11184788; doi:10.1186/s40164-024-00526-2)

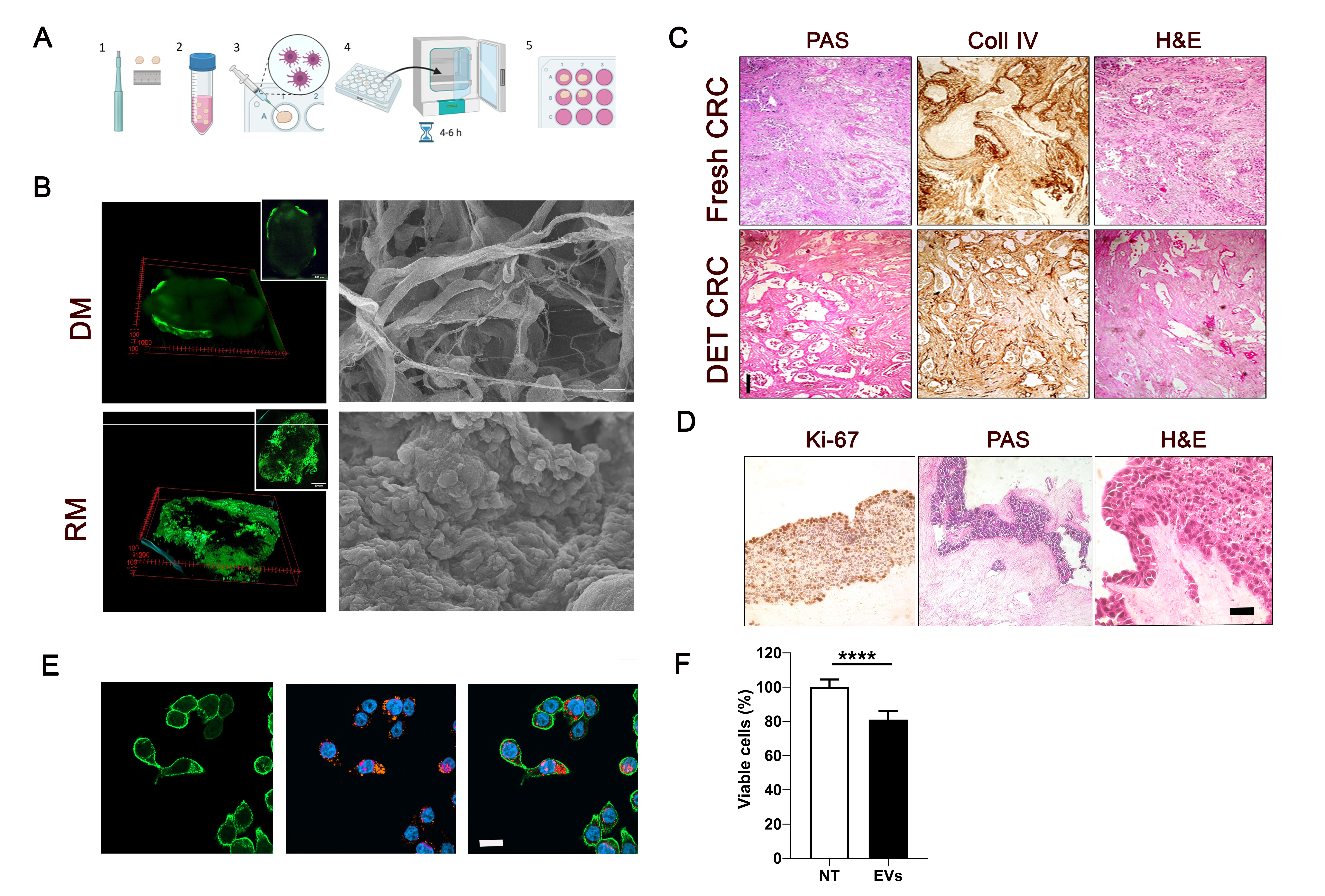

Supplement: Supplementary file 2 — Supplementary Material 2 [file 40164_2024_526_MOESM2_ESM.jpg]

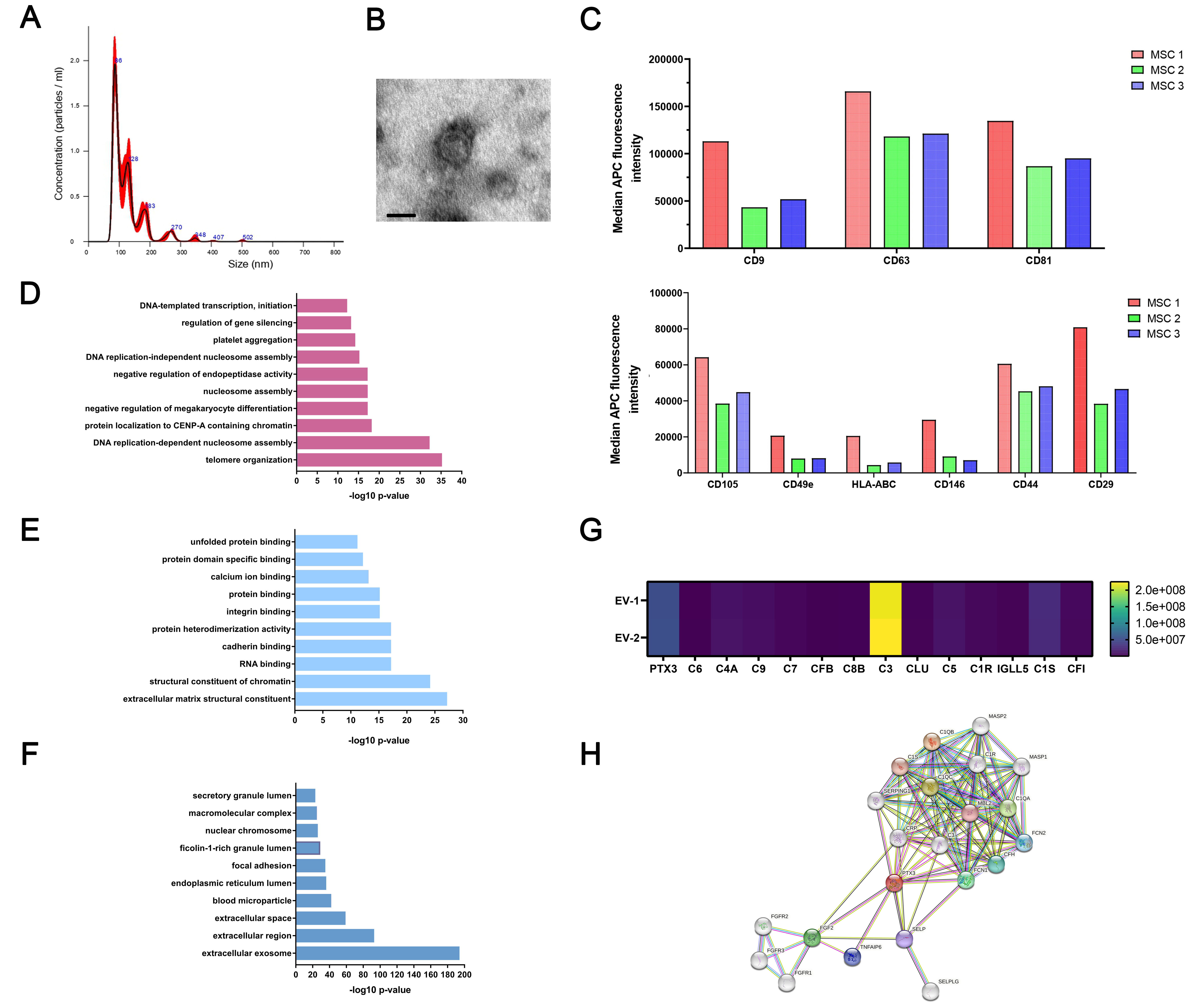

Supplement: Supplementary file 3 — Supplementary Material 3 [file 40164_2024_526_MOESM3_ESM.jpg]
